# Two New 7/7/6/5‐Tetracyclic C25 Steroids From the Marine‐Derived Fungus *Lodderomyces* sp. Z‐1

**DOI:** 10.1002/cbdv.71867

**Published:** 2026-10-22

**Authors:** Qi Zhang, Song‐Wei Li, Si‐Min Pan, Li‐Gong Yao, Yue‐Wei Guo, Ming‐Zhi Su

**Affiliations:** ^1^ School of Pharmacy Anhui University of Chinese Medicine Hefei Anhui China; ^2^ Shandong Laboratory of Yantai Drug Discovery Bohai Rim Advanced Research Institute for Drug Discovery Yantai Shandong China; ^3^ School of Medicine Shanghai University Shanghai China

**Keywords:** anti‐inflammatory activity, C25 steroids, fungus, *Lodderomyces*, starfish

## Abstract

*Lodderomyces* is recognized as a yeast genus with important applications in both food fermentation and medical research. In the present study, two new C25 steroids with a 7/7/6/5‐tetracyclic scaffold, named (11*R*,20*R*)‐11‐hydroxy‐24‐oxocyclocitrinol (**1**) and (11*R*)‐11‐hydroxy‐erythro‐23‐*O*‐methylneocyclocitrinol D (**2**), together with nine known analogues (**3–11**), were isolated and characterized from the marine‐derived fungus *Lodderomyces* sp. Z‐1, which was obtained from the starfish *Culcita novaeguineae* collected from the South China Sea. The structures and absolute configurations of new compounds were established through extensive spectroscopic analyses, quantum chemical calculations, and comparison of spectral data with those of related analogues reported in the literature. Additionally, the single‐crystal x‐ray crystallographic structure of the known compound **4** is reported here for the first time. In the anti‐inflammatory assay, compound **10** exhibited weak inhibitory activity against LPS‐induced NO production in RAW264.7 macrophages with an IC_50_ value of 41.72 ± 1.81 µM.

## Graphical abstract

Two new 7/7/6/5‐tetracyclic C25 steroids (**1** and **2**) and nine known analogues (**3**–**11**) were isolated and characterized from the starfish‐derived fungus *Lodderomyces* sp. Z‐1, with compound **10** showing weak anti‐inflammatory activity.

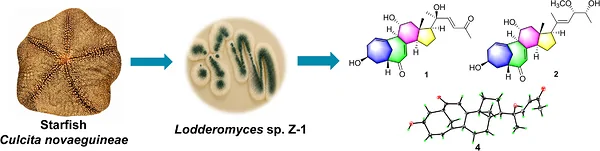

## 1 Introduction

Marine‐derived fungi have attracted considerable attention as important sources of chemically diverse natural products, as their adaptation to marine environments and host‐associated niches may promote the production of distinctive secondary metabolites [1, 2, 3, 4]. Among them, fungi associated with marine invertebrates represent particularly promising sources of new metabolites [5].

Classical steroids are characterized by a 6/6/6/5‐tetracyclic scaffold, whereas naturally occurring steroids with skeletal rearrangements represent structurally intriguing deviations from this conventional framework [6, 7]. Among these, cyclocitrinol‐like C25 steroids stand out with an unusual 7/7/6/5‐tetracyclic scaffold, whose structural diversity is governed by subtle modifications, particularly within the A/B ring system and side chains at C‐17 [8]. Interestingly, these C25 steroids have been shown to exhibit a spectrum of bioactivities, including antibacterial properties, moderate cytotoxicity against various cancer cell lines, and anti‐osteoporotic effects [9, 10, 11]. Classic steroidal compounds, such as dexamethasone, are well‐established for their potent anti‐inflammatory activities. However, the anti‐inflammatory potential of these structurally distinctive C25 steroids remains largely unexplored.

As documented in the literature, cyclocitrinol‐like C25 steroids have been predominantly isolated from fungi of the genus *Penicillium*, with sporadic reports from *Aspergillus* sp., *Chaetomium* sp., and the deep‐sea‐derived fungus *Rhizopus* sp. W23. Collectively, these findings suggest that the biosynthetic capacity for constructing the unusual 7/7/6/5‐tetracyclic scaffold from conventional 6/6/6/5‐tetracyclic (e.g., ergosterol) may be more broadly distributed across the fungal kingdom than previously recognized [8, 9, 10, 11, 12, 13].

The genus *Lodderomyces* belongs to the family *Debaryomycetaceae* and forms a monophyletic lineage closely related to the genera *Spathaspora* and *Scheffersomyces* [14]. Among the members of this genus, *L. elongisporus*, for example, has been reported to exhibit pronounced thermotolerance and high osmotolerance, enabling its survival under extreme environmental conditions. These robust physiological traits underscore its significant potential for application in the fermentation industry [15]. Moreover, the secondary metabolites of the genus *Lodderomyces* have been rarely investigated, and their chemical diversity remains largely unexplored.

As part of our ongoing research for new bioactive natural products from marine resources, a halotolerant fungal strain belonging to this genus, *Lodderomyces* sp. Z‐1, was isolated from a freshly collected specimen of the starfish *Culcita novaeguineae*. Chemical investigation of *Lodderomyces* sp. Z‐1 led to the isolation and characterization of two new 7/7/6/5‐tetracyclic C25 steroids, namely (11*R*,20*R*)‐11‐hydroxy‐24‐oxocyclocitrinol (**1**) and (11*R*)‐11‐hydroxy‐erythro‐23‐O‐methylneocyclocitrinol D (**2**), together with nine known analogues (**3**–**11**). Herein, we describe the cultivation, extraction, isolation, and structural elucidation of the secondary metabolites from this strain, as well as the evaluation of their anti‐inflammatory activities in vitro.

## 2 Results and Discussion

The ethyl acetate extract of the 32 L fermentation broth of *Lodderomyces* sp. Z‐1, a fungal strain isolated from the starfish *C. novaeguineae*, was subjected to repeated column chromatography, yielding eleven compounds. Among them, the known compounds **3**–**11** were identified as (20*R*)‐24‐oxocyclocitrinol (**3**) [11], (20*S*)‐24‐oxocyclocitrinol (**4**) [8], 22‐acetylisocyclocitrinol A (**5**) [9], neocyclocitrinols A–D (**6**–**9**) [8], erythro‐23‐*O*‐methylneocyclocitrinol (**10**) [8], and a known but previously unnamed compound, designated herein as 20‐*oxo*‐22,23,24,25‐tetranorcyclocitrinol (**11**) [16], respectively, by comparison of their ^1^H and ^13^C NMR spectroscopic data with those reported in literature.

Compound **1** was obtained as a colorless oil. Its molecular formula was established as C_25_H_34_O_5_ based on the HRESIMS ion peak at *m/z* 415.2485 [M + H]^+^ (calcd for 415.2479), corresponding to nine degrees of unsaturation. The ^13^C nuclear magnetic resonance (NMR) data (Table 1) revealed resonances for six sp^2^ olefinic (*δ*_C_ 157.6, 156.8, 144.5, 127.6, 126.8, and 126.1) and two carbonyl carbons (*δ*_C_ 207.2, 201.6), suggesting that **1** possesses a tetracyclic scaffold. A detailed comparison of the ^1^H and ^13^C NMR data revealed that compound **1** closely resembled the co‐occurring known compound (20*R*)‐24‐oxocyclocitrinol (**3**) [11], except that the C‐11 methylene group in **3** was replaced by a hydroxylated methine carbon resonating at *δ*_C_ 69.2 in **1**, suggesting that compound **1** is the C‐11 hydroxylated derivative of **3**, as supported by their 16 Da mass difference. Furthermore, the hydroxy group was assigned to C‐11 based on the ^1^H–^1^H correlation spectroscopy (COSY) correlations establishing the H‐9/H‐11/H‐12 spin system, together with the heteronuclear multiple‐bond correlation (HMBC) from H‐11 to C‐10 (Figure 2). Thus, the planar structure of compound **1** was established as shown in Figure 2.

**TABLE 1:** ^1^H and ^13^C NMR spectroscopic data for compounds **1** and **2** in CD_3_OD.^a^

No.	1		2	
	*δ*_C_, type	*δ*_H_ mult (*J* in Hz)	*δ*_C_, type	*δ*_H_ mult (*J* in Hz)
1	126.1, CH	5.64, dd (8.3, 6.3)	126.2, CH	5.66, m
2	37.1, CH_2_	2.54, m	37.1, CH_2_	2.55, m
		2.27, m		2.25, ov
3	65.2, CH	3.35, m	65.2, CH	3.37, m
4	42.2, CH_2_	2.84, m	42.3, CH_2_	2.85, m
		1.67, ov		1.68, ov
5	49.9, CH	2.78, m	49.9, CH	2.79, m
6	207.2, C	—	207.2, C	—
7	126.8, CH	5.56, s	126.5, CH	5.59, s
8	156.8, C	—	156.9, C	—
9	63.8, CH	2.64, m	64.0, CH	2.73, d (9.7)
10	144.5, C	—	144.4, C	—
11	69.2, CH	3.85, td (10.0, 5.0)	69.5, CH	3.92, m
12	48.8, CH_2_	2.27, m	48.1, CH_2_	2.25, ov
		1.33, m		1.47, m
13	47.0, C	—	47.8, C	—
14	56.8, CH	2.27, m	56.2, CH	2.41, m
15	22.8, CH_2_	2.02, m	23.6, CH_2_	1.68, 2H, ov
		1.92, ov		
16	23.2, CH_2_	1.67, ov	25.3, CH_2_	1.99, m
		1.54, m		1.82, m
17	60.2, CH	1.92, ov	60.6, CH	2.46, m
18	28.4, CH_2_	2.64, m	28.4, CH_2_	2.63, 2H, m
		2.54, m		
19	15.5, CH_3_	0.77, s	15.2, CH_3_	0.63, s
20	75.0, C	—	141.3, C	—
21	29.4, CH_3_	1.29, s	18.9, CH_3_	1.79, s
22	157.6, CH	7.08, d (15.9)	125.2, CH	5.24, d (9.1)
23	127.6, CH	6.33, d (15.9)	82.8, CH	3.96, dd (9.1, 3.8)
24	201.6, C	—	70.6, CH	3.78, m
25	27.3, CH_3_	2.32, s	18.5, CH_3_	1.11, d (6.5)
OMe			56.6, CH_3_	3.29, s

^a^ ^1^H NMR at 600 MHz, ^13^C NMR at 150 MHz; values are reported in ppm referenced to CD_3_OD (*δ*_H_ 3.31 and *δ*_C_ 49.0); assignments were aided by DEPT, HSQC, HMBC, and COSY experiments. Multiplicity abbreviations: “s” singlet, “d” doublet, “t” triplet, “m” multiplet, and “ov” overlapped.

The *E* geometry of the double bond Δ^22,23^ was assigned by the large coupling constants between H‐22 (*δ*_H_ 7.08, *J* = 15.9) and H‐23 (*δ*_H_ 6.33, *J* = 15.9). The highly similar NMR data between compounds **1** and **3** suggested that these two compounds shared the same stereochemistry in their core skeleton. In addition, the obvious nuclear overhauser effect spectroscopy (NOESY) correlation between H_3_‐19 and H‐11 indicated that these protons were oriented on the same face (Figure 2), thereby establishing the 11R* configuration of the newly generated C‐11 stereogenic center. However, the relative configuration at C‐20 remained ambiguous due to the flexibility of the side chain. To address this, GIAO NMR calculations and DP4+ analysis were performed. The ^13^C‐only analysis favored the 20R* epimer (96.56%), whereas the ^1^H‐only DP4+ analysis strongly favored the 20S* epimer (99.83%) (Figure S1.10). These contradictory results indicate that the relative configuration at C‐20 could not be unambiguously determined by computational methods alone, as such approaches may yield erratic outcomes when the chemical shift differences between isomers fall below the precision limit of the method [17]. To provide further evidence, the NMR data around C‐20 were compared with those of model compounds **3** and **4**. The data for compound **1** matched those of compound **3** more closely, particularly for H‐21 and C‐22 (Table S1). Accordingly, compound **1** was assigned the same 20R* configuration as compound **3**.

The absolute configuration of compound **1** was further established as 3*S*,5*S*,9*R*,11*R*,13*S*,14*R*,17*S*,20*R* based on the good agreement between the time‐dependent density functional theory‐electronic circular dichroism (TDDFT‐ECD) calculated and experimental ECD spectra (Figure 3). This assignment was further corroborated by the x‑ray crystallographic analysis of the co‑occurring compound **4**, for which single crystals were obtained for the first time in this study (Figure 4, CCDC NO. 2576293). The x‐ray data unambiguously assigned the absolute configuration of **4** as 3*S*,5*S*,9*R*,13*S*,14*R*,17*S*,20*S* [Flack parameter = 0.01(12)], providing solid support for the configurational assignments of the core skeleton of this structural class. Accordingly, compound **1** was determined to be the 11α‐hydroxy derivative of (20*R*)‐24‐oxocyclocitrinol (**3**), as depicted in Figure 1, and was designated (11*R*,20*R*)‐11‐hydroxy‐24‐oxocyclocitrinol.

**FIGURE 1:**
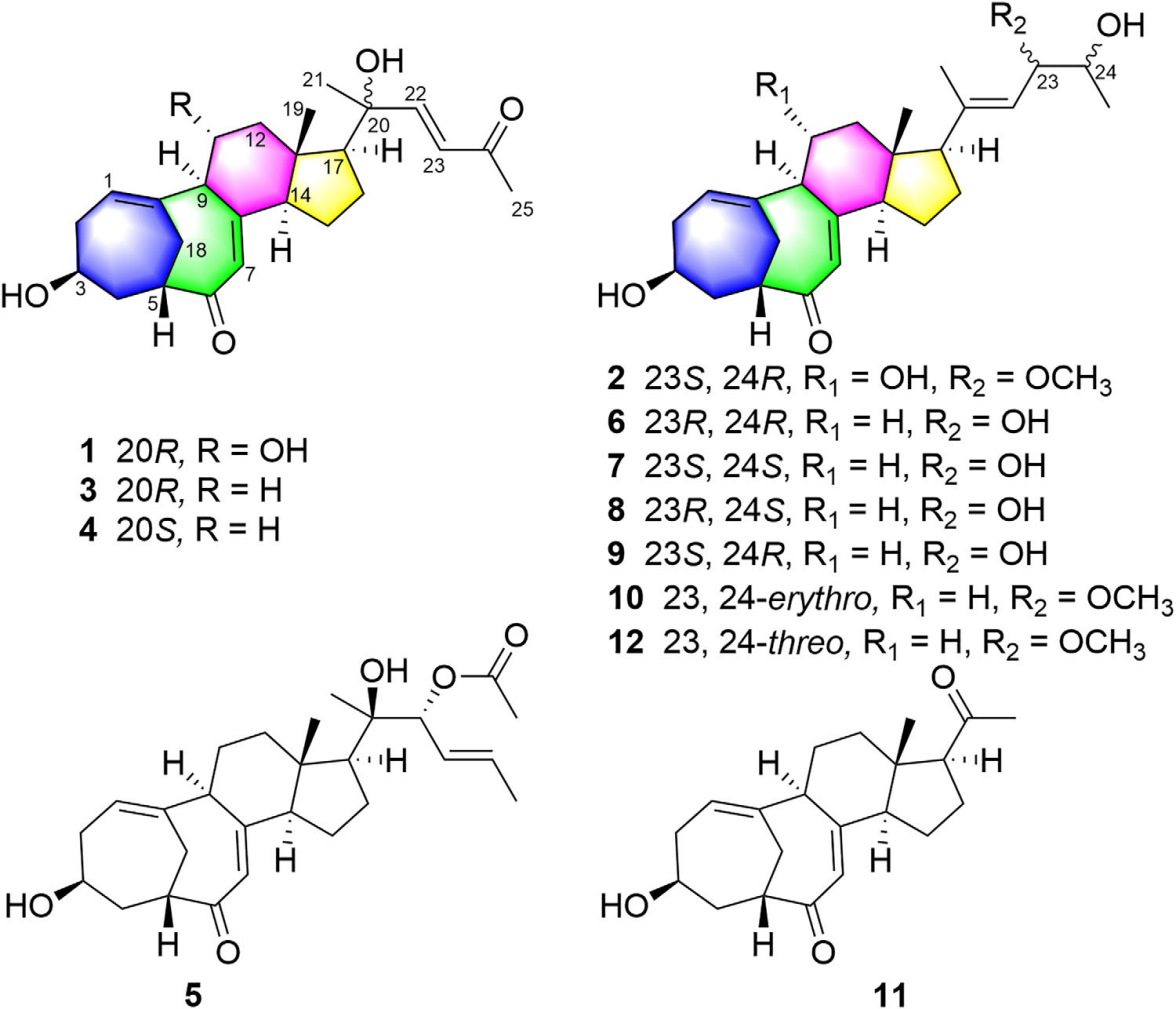
Chemical structures of compounds **1**–**12**.

Compound **2** was obtained as a colorless oil. Its molecular formula was established as C_26_H_38_O_5_ based on the HRESIMS ion peak at *m/z* 431.2796 [M + H]^+^ (calcd. for 431.2791), corresponding to eight degrees of unsaturation. The ^13^C NMR data (Table 1) revealed resonances for six sp^2^ olefinic carbons (*δ*_C_ 156.9, 144.4, 141.3, 126.5, 126.2, 125.2) and one carbonyl carbon (*δ*_C_ 207.2), suggesting that compound **2** incorporates four rings. The ^1^H and ^13^C NMR data (Table 1) of **2** closely resembled those of erythro‐23‐*O*‐methylneocyclocitrinol (**10**), suggesting that both compounds shared the same carbon skeleton. The principal difference was the presence of an oxygenated methine resonating at *δ*_C_ 69.5 in **2**, instead of the corresponding non‐oxygenated aliphatic carbon in **10**. The location of this oxygenated methine at C‐11 was supported by the ^1^H–^1^H COSY correlations of H‐11 with H‐9 and H‐12 and the HMBC correlations from H‐11 to C‐10 (Figure 2). Therefore, the planar structure of **2** was established.

**FIGURE 2:**
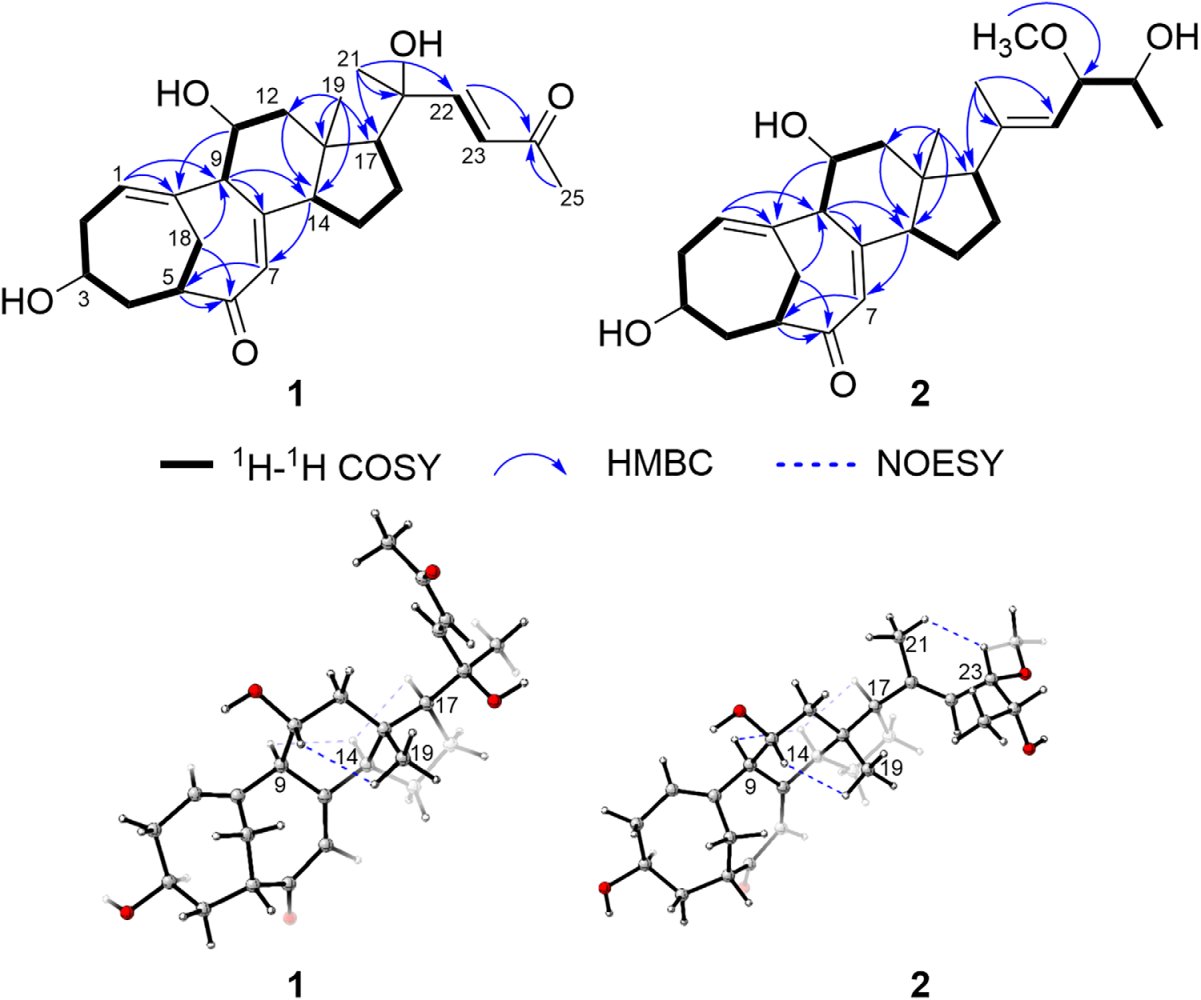
^1^H–^1^H COSY, key HMBC and NOESY correlations of compounds **1** and **2**.

**FIGURE 3:**
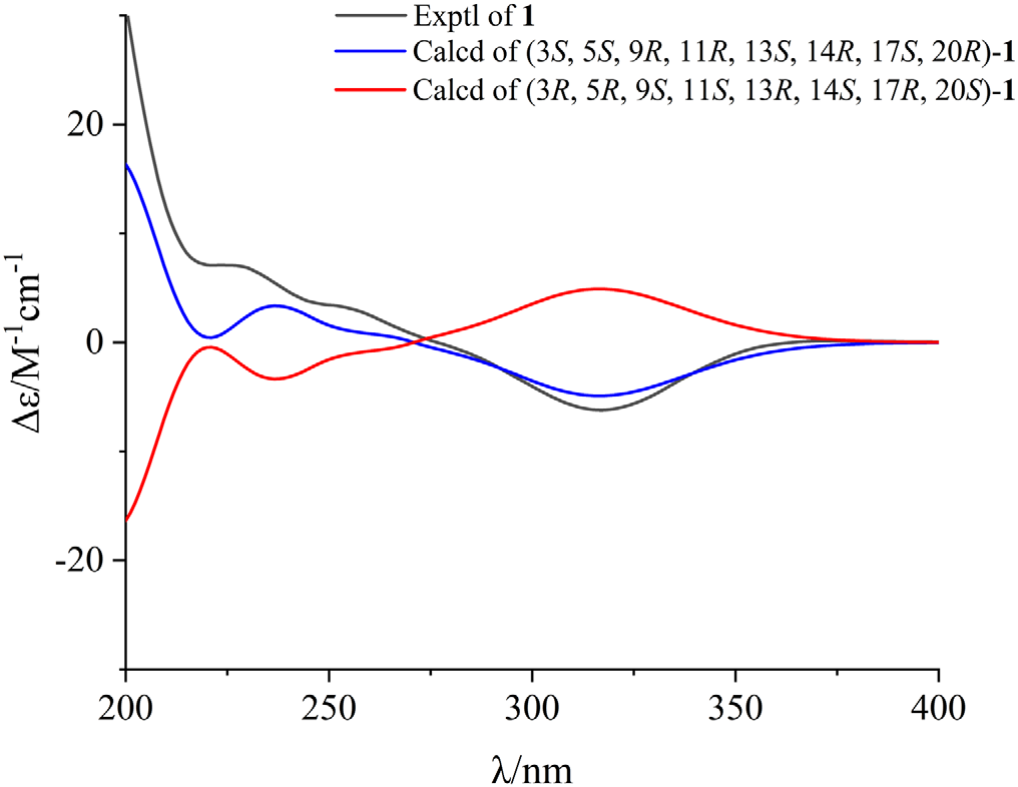
The experimental ECD spectrum of compound **1** (black) matched well with the calculated spectrum (blue) and showed opposite Cotton effects to the mirror‑‐image curve (red).

**FIGURE 4:**
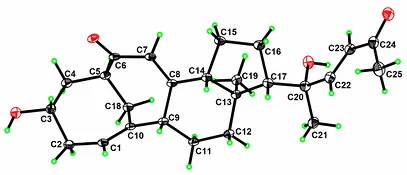
X‐ray crystallographic structure of compound **4**.

The *E* geometry of the Δ^20,22^ double bond was supported by the NOESY correlation between H_3_‐21 (*δ*_H_ 1.79) and H‐23 (*δ*_H_ 3.96), together with the absence of a NOESY cross‐peak between H_3_‐21 and H‐22. The relative configuration of the steroidal nucleus was assigned by comparison with structurally related C25 steroids **6**–**10** and **12** [8], whose ring‐junction stereochemistry is generally conserved. This assignment was supported by the NOESY correlations (Figure 2) of H_3_‐19/H‐11, H‐9/H‐14, and H‐14/H‐17, indicating that H‐11, H_3_‐19, H‐9, H‐14, and H‐17 were cofacial, thereby establishing the 11R* configuration of the newly generated C‐11 stereogenic center. The vicinal coupling constant between H‐23 and H‐24 in **2** (*J*_23,24_ = 3.8 Hz) was consistent with the *erythro* relationship between C‐23 and C‐24 in **10** (*J*_23,24_ = 4.1 Hz), rather than the *threo* relationship observed in **12** (*J*_23,24_ = 6.4 Hz) [8]. However, considering the conformational flexibility of the side chain, this coupling constant alone was not regarded as sufficient to exclude the two possible *threo* candidates. Therefore, to establish the complete relative configuration of **2**, GIAO NMR calculations followed by DP4+ probability analysis were performed for all four possible C‐23/C‐24 diastereomers. DP4+ analysis showed that the results assigned a probability exceeding 99% to isomer 2, corresponding to the 3S*,5S*,9R*,11R*,13R*,14R*,17R*,23S*,24R* diastereomer (Figure S2.10), thereby supporting the determination of the relative configuration of compound **2**.

Similarly, the TDDFT‐ECD calculated curve matched well with the experimental ECD spectrum of compound **2** (Figure 5), allowing the absolute configuration of **2** to be assigned as 3*S*,5*S*,9*R*,11*R*,13*R*,14*R*,17*R*,23*S*,24*R*. Finally, compound **2** was determined to be the 11*α*‐hydroxy derivative of erythro‐23‐*O*‐methylneocyclocitrinol (**10**), as depicted in Figure 1, and was designated (11*R*)‐11‐hydroxy‐erythro‐23‐*O*‐methylneocyclocitrinol D.

**FIGURE 5:**
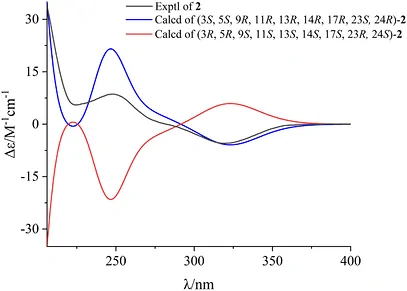
Comparison of the experimental (black) and calculated ECD spectra (blue and red) of compound **2**.

In vitro, all isolated compounds (**1**–**11**) were evaluated for their anti‐inflammatory activity by measuring their inhibitory effects on LPS‐induced NO production in RAW264.7 macrophages. Among them, compound **10** exhibited weak inhibitory activity, with an IC_50_ value of 41.72 ± 1.81 µM, compared with the positive control dexamethasone (IC_50_ = 1.24 ± 0.41 µM), whereas the other compounds did not display significant NO inhibition up to 50 µM.

To exclude the possibility that the observed activity was due to cytotoxicity, the cell viability of compounds **1**–**11** was assessed using the CCK‐8 assay at 50 µM. Compound **10** inhibited NO production by 61.81% while retaining 83.99% relative cell viability. In contrast, compound **4** reduced relative cell viability to 49.65%, indicating a cytotoxic effect at this concentration, whereas the remaining compounds retained 75.46%–98.11% relative cell viability (Table S2).

## 3 Conclusions

In summary, this is the first study to report the isolation and identification of 7/7/6/5‐tetracyclic C25 steroids from a starfish‐derived fungus *Lodderomyces* sp., yielding two new compounds, (11*R*,20*R*)‐11‐hydroxy‐24‐oxocyclocitrinol (**1**) and (11*R*)‐11‐hydroxy‐erythro‐23‐*O*‐methylneocyclocitrinol D (**2**), alongside nine known compounds (**3**–**11**). The structures and absolute configurations of the new compounds were established through a combination of extensive NMR analyses, DP4+ probability analysis, and TDDFT‐ECD calculations. Notably, this study also reports the first x‐ray crystallographic characterization of known compound **4**, which provided independent evidence for the configurational assignments of this class of steroids. Bioassay results revealed that compound **10** possesses weak inhibitory activity against LPS‐induced NO production in RAW264.7 macrophages, with an IC_50_ value of 41.72 ± 1.81 µM.

Although compound **10** exhibited only weak anti‐inflammatory activity, its therapeutic potential in other diseases such as antimicrobial and metabolic disorders remains to be explored, particularly given the constraints of the current screening platform. Notably, this study provides the first evidence of cyclocitrinol‐type C25 steroids from a *Lodderomyces* fungus, thereby broadening the source diversity of this structurally distinctive class of steroids. Collectively, these results not only expand the chemical repertoire of C25 steroids but also underscore the view that starfish‐associated marine‐derived *Lodderomyces* sp. represent a promising reservoir of structurally diverse and pharmaceutically relevant secondary metabolites.

## 4 Experimental Section

### 4.1 General Experimental Procedures

Optical rotations were measured on an Anton Paar MCP 530 polarimeter (Anton Paar Trading Co., Shanghai, China). IR spectra were recorded using a Nicolet iS50 FT‐IR spectrometer (Thermo Fisher Scientific, Madison, WI, USA). ^1^H and ^13^C NMR spectra were acquired on a Bruker DRX‐600 spectrometer (Bruker BioSpin AG, Fällanden, Switzerland), using residual solvent signals as internal references: CD_3_OD (*δ*_H_ 3.31, *δ*_C_ 49.00). HRESIMS data were obtained on a ZenoTOF 7600 mass spectrometer (SCIEX). Silica gel for column chromatography was purchased from Qingdao Marine Chemical Co., Ltd. (Qingdao, China), and Sephadex LH‐20 was supplied by Amersham Biosciences (Uppsala, Sweden). Thin‐layer chromatography (TLC) was performed on precoated silica gel plates (Silica Gel 60 GF254, Yantai Zifu Chemical Group Co., Ltd., Yantai, China), and the spots were visualized under UV light or by heating after spraying with vanillin–H_2_SO_4_ reagent. Reversed‐phase high‐performance liquid chromatography (RP‐HPLC) was performed on an Agilent 1260 system equipped with a DAD G1315D detector at 210 nm. Semi‐preparative HPLC separation was carried out using an Agilent XDB‐C18 column (5 µm, 250 × 9.4 mm), while an analytical XDB‐C18 column (5 µm, 250 × 4.6 mm) was used for HPLC analysis. Solvents used for column chromatography and HPLC were of analytical grade (Shanghai Chemical Reagent Co., Ltd., Shanghai, China) or chromatographic grade (Dikma Technologies Inc., Beijing, China).

### 4.2 Fungal Material and Fermentation

The fungal strain (*Lodderomyces* sp. Z‐1) used in this experiment was isolated from starfish *C. novaeguineae* (voucher specimen No. SYT‐17) collected near Ximao Island, Hainan Province, China, at a depth of 20 m, in June 2023, and identified by Xiu‐Bao Li from Hainan University. The fungal strain (No. Z‐1) is deposited at the Shandong Laboratory of Yantai Drug Discovery, Shandong, China, and maintained at −80°C. Taxonomic identification was performed by Sangon Biotech (Shanghai) Co., Ltd. BLAST analysis of the ITS sequence showed 99.10% similarity to *Lodderomyces elongisporus* (GenBank accession no. KU095861). The ITS sequence of strain Z‐1 was deposited in GenBank under accession number PZ902113. Accordingly, the strain was tentatively identified as *Lodderomyces* sp. and designated *Lodderomyces* sp. Z‐1.

*Lodderomyces* sp. Z‐1 was initially cultured on sterile medium (prepared with 75% seawater) containing glucose (20 g/L), malt extract (20 g/L), peptone (1 g/L), and agar (20 g/L) at 28°C in a biochemical incubator for 3 days. Subsequently, fermentation was carried out in 32 Erlenmeyer flasks (2 L capacity), each containing 1 L of the same medium without agar, on a rotary shaker at 220 rpm and 28°C for 21 days. After fermentation, the culture broth was extracted with ethyl acetate, and the combined organic phases were concentrated under reduced pressure to yield the crude extract.

### 4.3 Extraction and Isolation

The crude extract (30 g) was subjected to silica gel column chromatography and eluted with a petroleum ether–EtOAc gradient (1:0–0:1) to afford nine fractions (Fr. A–Fr. I). Fraction H (3.0 g) was further separated by silica gel column chromatography using a DCM–MeOH gradient (100:1–5:1) to yield six subfractions (Fr. HA–Fr. HF). Subfraction Fr. HE was purified by semi‐preparative HPLC to give compounds **1** (2.0 mg, *t*_R_ = 32 min, 28% CH_3_CN–H_2_O, 3 mL/min), **3** (2.8 mg, *t*_R_ = 40 min, 40% CH_3_CN–H_2_O, 3 mL/min), and **4** (4.6 mg, *t*_R_ = 78 min, 40% CH_3_CN–H_2_O, 3 mL/min).

Fraction I (3.0 g) was further separated by Sephadex LH‐20 column chromatography, eluted with MeOH to afford eight subfractions (Fr. IA–Fr. IH). Subfraction Fr. IE was purified by semi‐preparative HPLC to yield compound **2** (2.3 mg, *t*_R_ = 22 min, 27% CH_3_CN–H_2_O, 3 mL/min), together with compounds **5** (2.0 mg, *t*_R_ = 40 min), **6** (2.8 mg, *t*_R_ = 47 min), **7** (3.2 mg, *t*_R_ = 52 min), and **8** (1.8 mg, *t*_R_ = 56 min, 52% CH_3_CN–H_2_O, 3 mL/min). Subfraction Fr. IF (1.0 g) was separated by silica gel column chromatography using a DCM–MeOH gradient (50:1–4:1) to afford seven subfractions (Fr. IFA–Fr. IFG). Subfraction Fr. IFG was purified by semi‐preparative HPLC to give compound **10** (2.0 mg, *t*_R_ = 11 min, 23% CH_3_CN–H_2_O, 3 mL/min).

Fraction G (2.4 g) was separated by silica gel column chromatography using a DCM–MeOH gradient (100:1–5:1) to afford six subfractions (Fr. GA–Fr. GF). Subfraction Fr. GA was further separated by silica gel column chromatography using a DCM–MeOH gradient (100:1–20:1) to yield nine subfractions (Fr. GAA–Fr. GAI). Subfraction Fr. GAE was purified by semi‐preparative HPLC to give compound **9** (2.0 mg, *t*_R_ = 28 min, 23% CH_3_CN–H_2_O, 3 mL/min), and subfraction Fr. GAF was purified by semi‐preparative HPLC to give compound **11** (2.0 mg, *t*_R_ = 37 min, 18% CH_3_CN–H_2_O, 3 mL/min).

#### 4.3.1 (11*R*,20*R*)‐11‐Hydroxy‐24‐oxocyclocitrinol (**1**)

Colorless oil. ${\left[\alpha \right]}_{D}^{20}$ +144.0 (*c* 0.10 MeOH); UV (MeOH) *λ*_max_ (log *ε*) = 194 (4.62), 235 (4.57) nm; ECD (MeOH) *λ*_max_ (Δ*ε*) 316 (−6.2) nm; IR (KBr) *ν*_max_ 3413, 2922, 1648, 1459, 1029, 731 cm^−1^; ^1^H NMR (CD_3_OD, 600 MHz) and ^13^C NMR (CD_3_OD, 150 MHz) data, see Table 1; HR‐ESI‐MS *m/z* 415.2485 [M + H]^+^ (calcd. 415.2479).

#### 4.3.2 (11*R*)‐11‐Hydroxy‐erythro‐23‐*O*‐methylneocyclocitrinol D (**2**)

Colorless oil. ${\left[\alpha \right]}_{D}^{20}$ +120.0 (*c* 0.03 MeOH); UV (MeOH) *λ*_max_ (log *ε*) = 192 (3.73), 240 (3.31) nm; ECD (MeOH) *λ*_max_ (Δε) 218 (+5.8), 248 (+8.5), 320 (−5.5) nm; IR (KBr) *ν*_max_ 3405, 2928, 1643, 1261, 1030, 734 cm^−1^; ^1^H NMR (CD_3_OD, 600 MHz) and ^13^C NMR (CD_3_OD, 150 MHz) data, see Table 1; HR‐ESI‐MS *m/z* 431.2796 [M + H]^+^ (calcd. for 431.2791).

### 4.4 X‐Ray Diffraction Analysis

Single colorless plate‐shaped crystals of compound **4** were used as supplied. A suitable crystal with dimensions 0.154 × 0.095 × 0.022 mm^3^ was selected. Single‐crystal x‐ray diffraction data were collected by a Bruker D8 Venture diffractometer with Cu Kα radiation (*λ* = 1.54178 Å). The crystal was kept at a steady *T* = 170(2) K during data collection. The structure was solved with the ShelXT structure program using Intrinsic Phasing and refined with ShelXL using full‐matrix least‐squares minimisation on *F* ^2^.

Crystal data for compound **4**: C_25_H_34_O_4_•0.5(CH_4_O)•H_2_O, M = 432.56 g/mol, orthorhombic, space group *P*2_1_2_1_2_1_ (no. 19), *a* = 6.6903(3) Å, *b* = 20.7848(10) Å, *c* = 33.1374(13) Å, *α* = 90°, *β* = 90°, *γ* = 90°, *V* = 4608.0(4) Å3, *T* = 170(2) K, *Z* = 8, μ(Cu Kα) = 0.693 mm^−1^, 29715 reflections measured, 8732 independent reflections (Rint = 0.0729). The final R1 value was 0.0577 (I > 2σ(I)). The final wR(F2) value was 0.1586 (I > 2σ(I)). The final R1 value was 0.0746 (all data). The final wR(F2) value was 0.1751 (all data). The goodness of fit on *F* ^2^ was 1.038. Flack parameter = 0.01(12).

### 4.5 Calculation Details

Quantum chemical NMR and TDDFT‐ECD calculations were performed to assist in the assignment of the relative and absolute configurations of the isolated compounds.

For QM‐NMR calculations, conformational searches were carried out with MacroModel 9.233 (Schrödinger, http://www.schrodinger.com/MacroModel, accessed on 31 July 2025) using the Monte Carlo multiple minimum (MCMM) method and the OPLS_2005 force field within an energy window of 21 kJ/mol (5.0 kcal/mol). Conformers with Boltzmann populations greater than 1% were further optimized at the B3LYP/6‐31G(d,p) level. The NMR shielding constants of the optimized conformers were then calculated by the gauge‐independent atomic orbital (GIAO) method [18, 19, 20, 21] at the PCM/mPW1PW91/6‐311+G(d,p) level using Gaussian 09 [22]. The shielding constants were Boltzmann‐averaged and compared with the experimental NMR data using DP4+ probability analysis.

For ECD calculations, conformers with Boltzmann populations above 1% after geometry optimization were subjected to TDDFT calculations at the B3LYP/6‐31G(d,p) level with the IEFPCM solvent model in Gaussian 09. The calculated ECD spectra were generated using SpecDis 1.71 and compared with the experimental spectra [23].

### 4.6 Anti‐Inflammatory Assay

The anti‐inflammatory activity of compounds **1**–**11** was evaluated by measuring their inhibitory effects on LPS‐induced NO production in RAW264.7 macrophages. The murine macrophage cell line RAW264.7 (RRID: CVCL_0493) was obtained from Wuhan Pricella Biotechnology Co., Ltd. (Wuhan, China) and cultured in DMEM supplemented with 10% fetal bovine serum and 1% penicillin–streptomycin at 37°C in a humidified atmosphere containing 5% CO_2_. The cells (3 × 10^4^) were seeded into 96‐well plates and allowed to adhere overnight. The tested compounds were dissolved in DMSO and diluted with culture medium to concentrations of 3.12−50 µM, with the final DMSO concentration maintained below 0.1%. Dexamethasone was used as the positive control and tested in parallel under the same experimental conditions. After pretreatment with the compounds for 1 h, the cells were stimulated with LPS 50 ng/ml to induce NO production. After incubation for 24 h, 70 µL of culture supernatant was collected and mixed with an equal volume of Griess reagent. The absorbance was measured at 540 nm using a microplate reader (EnVision, PerkinElmer, Pontyclun, UK), and the nitrite concentration was calculated from a sodium nitrite standard curve.

### 4.7 Cytotoxicity Assay

The cytotoxicity of compounds **1**–**11** toward RAW264.7 cells was evaluated using the Cell Counting Kit‐8 (CCK‐8) assay under the same treatment conditions as the NO‐inhibition assay. RAW264.7 cells were seeded into 96‐well plates at a density of 3 × 10^4^ cells per well and allowed to adhere overnight. The cells were treated with each compound at 50 µM for 24 h; CCK‐8 reagent was added to each well, and the plates were incubated at 37°C according to the manufacturer's instructions. Absorbance was measured at 450 nm using a microplate reader. Each compound was tested in three replicate wells within a single experiment.

### 4.8 Statistical Analysis

Each assay was conducted once with three replicate wells per condition. NO inhibition rates and relative cell viability at the specified test concentrations are reported as the mean ± standard deviation (SD) of three technical replicate wells. For IC_50_ determination, three technical replicate dose–response curves were fitted separately by nonlinear regression using GraphPad Prism. The resulting IC_50_ values are presented as the mean ± SD of the three fitted estimates. No inferential statistical tests were performed.

## Author Contributions

Conceptualization, M.‐Z. Su and Y.‐W. Guo; funding acquisition, Y.‐W. Guo; methodology, Y.‐W. Guo; chemical investigation, Q. Zhang; bioactivity assays, S.‐M. Pan; collection of marine material, L.‐G. Yao; Writing – original draft preparation, Q. Zhang; Writing – review and editing, S.‐W. Li and M.‐Z. Su. All authors have read and agreed to the published version of the manuscript.

## Conflicts of Interest

The authors declare no conflicts of interest.

## Supporting information




**Supporting File 1**: cbdv71867‐sup‐0001‐SuppMat.docx.

## Data Availability

The data that support the findings of this study are available in the Supporting Information, which includes the following: NMR, HRESIMS, and IR spectra and DP4+ results for compounds **1** and **2** (Figures S1.1–S1.10 and S2.1–S2.10); ^1^H and ^13^C NMR spectra of compounds **3**–**11** (Figures S3.1, S3.2, S4.1, S4.2, S5.1, S5.2, S6.1, S6.2, S7.1, S7.2, S8.1, S8.2, S9.1, S9.2, S10.1, S10.2, S11.1, and S11.2, respectively); a comparison of the NMR data around C‐20 of compound **1** with those of compounds **3** and **4** (Table S1); the ITS sequence of *Lodderomyces* sp. Z‐1; and NO inhibition and relative cell viability data for compounds **1**–**11** in RAW264.7 cells (Table S2).
