# Supplementary material for: Two New 7/7/6/5‐Tetracyclic C25 Steroids From the Marine‐Derived Fungus Lodderomyces sp. Z‐1
Source: Chem Biodivers. 2026 Oct 22;23(10):e71867. doi: 10.1002/cbdv.71867 (PMC13687916; doi:10.1002/cbdv.71867)
Supplement: Supplementary file 1 [file CBDV-23-e71867-s001.docx]

***Supporting Information for***

**Two New 7/7/6/5-Tetracyclic C25 Steroids from the Marine-derived Fungus *Lodderomyces* sp. Z-1**

Qi Zhang,^a,b^^,1^ Song-Wei Li,^c,1^ Si-Min Pan,^b^ Li-Gong Yao,^b^ Yue-Wei Guo,*^,b^ Ming-Zhi Su*^,a,b^

^a^ School of Pharmacy, Anhui University of Chinese Medicine, Hefei Anhui 230012, China

^b^ Shandong Laboratory of Yantai Drug Discovery, Bohai Rim Advanced Research Institute for Drug Discovery, Yantai, Shandong 264117, China, e-mails: mzsu@baridd.ac.cn (M.-Z. Su), [ywguo@simm.ac.cn](mailto:ywguo@simm.ac.cn) (Y.-W. Guo)

^c^ School of Medicine, Shanghai University, Shanghai 200444, China

*Corresponding authors：Yue-Wei Guo ([ywguo@simm.ac.cn](mailto:ywguo@simm.ac.cn)), Ming-Zhi Su (mzsu@baridd.ac.cn)

^1^ These authors contributed equally to this work.

**Contents**

[1. Spectra of compound **1**	4](#_Toc23573)

[Figure S1.1 ^1^H NMR spectrum (600 MHz) of compound **1** in CD_3_OD	4](#_Toc31291)

[Figure S1.2 ^13^C NMR spectrum (150 MHz) of compound **1** in CD_3_OD	4](#_Toc32472)

[Figure S1.3 DEPT spectrum (150 MHz) of compound **1** in CD_3_OD	5](#_Toc5701)

[Figure S1.4 HSQC spectrum (600 MHz) of compound **1** in CD_3_OD	5](#_Toc22748)

[Figure S1.5 HMBC spectrum (600 MHz) of compound **1** in CD_3_OD	6](#_Toc19570)

[Figure S1.6 ^1^H-^1^H COSY spectrum (600 MHz) of compound **1** in CD_3_OD	6](#_Toc32210)

[Figure S1.7 NOESY spectrum (600 MHz) of compound **1** in CD_3_OD	7](#_Toc19129)

[Figure S1.8 HR-ESIMS spectrum of compound **1**	7](#_Toc28828)

[Figure S1.9 IR spectrum of compound **1**	8](#_Toc2589)

[Figure S1.10 DP4+ results of compound **1** (Isomer 1: 3*S**, 5*S**, 9*R**, 11*R**, 13*S**, 14*R**, 17*S**, 20*S**; Isomer 2: 3*S**, 5*S**, 9*R**, 11*R**, 13*S**, 14*R**, 17*S**, 20*R**).	8](#_Toc18765)

[Table S1. Comparison of the NMR data around C-20 of compound **1** with those of **3** and **4**.	8](#_Toc8468)

[2. Spectra of compound **2**	9](#_Toc28781)

[Figure S2.1 ^1^H NMR spectrum (600 MHz) of compound **2** in CD_3_OD	9](#_Toc31303)

[Figure S2.2 ^13^C NMR spectrum (150 MHz) of compound **2** in CD_3_OD	9](#_Toc16698)

[Figure S2.3 DEPT spectrum (150 MHz) of compound **2** in CD_3_OD	10](#_Toc6735)

[Figure S2.4 HSQC spectrum (600 MHz) of compound **2** in CD_3_OD	10](#_Toc13041)

[Figure S2.5 HMBC spectrum (600 MHz) of compound **2** in CD_3_OD	11](#_Toc13401)

[Figure S2.6 ^1^H-^1^H COSY spectrum (600 MHz) of compound **2** in CD_3_OD	11](#_Toc26984)

[Figure S2.7 NOESY spectrum (600 MHz) of compound **2** in CD_3_OD	12](#_Toc3281)

[Figure S2.8 HR-ESIMS spectrum of compound **2**	12](#_Toc20359)

[Figure S2.9 IR spectrum of compound **2**	13](#_Toc25222)

[Figure S2.10 DP4+ results of compound **2** (Isomer 1: 3*S**, 5*S**, 9*R**, 11*R**, 13*R**, 14*R**, 17*R**, 23*R**, 24*S**; Isomer 2: 3*S**, 5*S**, 9*R**, 11*R**, 13*R**, 14*R**, 17*R**, 23*S**, 24*R**; Isomer 3: 3*S**, 5*S**, 9*R**, 11*R**, 13*R**, 14*R**, 17*R**, 23*R**, 24*R**; Isomer 4: 3*S**, 5*S**, 9*R**, 11*R**, 13*R**, 14*R**, 17*R**, 23*S**, 24*S**)	13](#_Toc12886)

[3. Spectra of compound **3**	14](#_Toc6637)

[Figure S3.1 ^1^H NMR spectrum (600 MHz) of compound **3** in CD_3_OD	14](#_Toc18881)

[Figure S3.2 ^13^C NMR spectrum (150 MHz) of compound **3** in CD_3_OD	14](#_Toc870)

[4. Spectra of compound **4**	15](#_Toc17478)

[Figure S4.1 ^1^H NMR spectrum (600 MHz) of compound **4** in CDCl3	15](#_Toc7341)

[Figure S4.2 ^13^C NMR spectrum (150 MHz) of compound **4** in CDCl3	15](#_Toc21785)

[5. Spectra of compound **5**	16](#_Toc28385)

[Figure S5.1 ^1^H NMR spectrum (600 MHz) of compound **5** in CD_3_OD	16](#_Toc146)

[Figure S5.2 ^13^C NMR spectrum (150 MHz) of compound **5** in CD_3_OD	16](#_Toc30158)

[6. Spectra of compound **6**	17](#_Toc223)

[Figure S6.1 ^1^H NMR spectrum (600 MHz) of compound **6** in DMSO-*d*_6_	17](#_Toc8783)

[Figure S6.2 ^13^C NMR spectrum (150 MHz) of compound **6** in DMSO-*d*_6_	17](#_Toc11587)

[7. Spectra of compound **7**	18](#_Toc22795)

[Figure S7.1](#_Toc24694) ^[1](#_Toc24694)^[H NMR spectrum (600 MHz) of compound](#_Toc24694) **[7](#_Toc24694)** [in CD](#_Toc24694)_[3](#_Toc24694)_[OD	18](#_Toc24694)

[Figure S7.2](#_Toc24694) ^[13](#_Toc24694)^[C NMR spectrum (150 MHz) of compound](#_Toc24694) **[7](#_Toc24694)** [in CD](#_Toc24694)_[3](#_Toc24694)_[OD	18](#_Toc24694)

[8. Spectra of compound **8**	19](#_Toc6109)

[Figure S8.1 ^1^H NMR spectrum (600 MHz) of compound **8** in CD_3_OD	19](#_Toc32480)

[Figure S8.2 ^13^C NMR spectrum (150 MHz) of compound **8** in CD_3_OD	19](#_Toc32671)

[9. Spectra of compound **9**	20](#_Toc960)

[Figure S9.1 ^1^H NMR spectrum (600 MHz) of compound **9** in CD_3_OD	20](#_Toc11571)

[Figure S9.2 ^13^C NMR spectrum (150 MHz) of compound **9** in CD_3_OD	20](#_Toc29828)

[10. Spectra of compound **10**	21](#_Toc25605)

[Figure S10.1 ^1^H NMR spectrum (600 MHz) of compound **10** in DMSO-*d*_6_	21](#_Toc21463)

[Figure S10.2 ^13^C NMR spectrum (150 MHz) of compound **10** in DMSO-*d*_6_	21](#_Toc23892)

[11. Spectra of compound **11**	22](#_Toc14899)

[Figure S11.1 ^1^H NMR spectrum (600 MHz) of compound **11** in CD_3_OD	22](#_Toc27394)

[Figure S11.2 ^13^C NMR spectrum (150 MHz) of compound **11** in CD_3_OD	22](#_Toc10238)

[12. ITS Sequence of *Lodderomyces* sp. Z-1	23](#_Toc20933)

[13. NO Inhibition and Cell Viability	23](#_Toc1390)

[Table S2. Inhibition of LPS-induced NO production and relative cell viability in RAW264.7 cells treated with compounds **1**–**11** at 50 μM	23](#_Toc7653)

# 1. Spectra of compound 1

## **Figure S1.1** ^1^H NMR spectrum (600 MHz) of compound **1** in CD_3_OD

## **Figure S1.2** ^13^C NMR spectrum (150 MHz) of compound **1** in CD_3_OD

 **Figure S1.3** DEPT spectrum (150 MHz) of compound **1** in CD_3_OD

## **Figure S1.4** HSQC spectrum (600 MHz) of compound **1** in CD_3_OD

## **Figure S1.5** HMBC spectrum (600 MHz) of compound **1** in CD_3_OD

## **Figure S1.6** ^1^H-^1^H COSY spectrum (600 MHz) of compound **1** in CD_3_OD

## **Figure S1.7** NOESY spectrum (600 MHz) of compound **1** in CD_3_OD

**Formula Calculator Results**

| Measured m/z | Cal m/z | Error(mmu) | Error(ppm) | Ion Formula | Ion |
| --- | --- | --- | --- | --- | --- |
| 415.2485 | 415.2479 | 0.6 | 1.5 | C25H35O5 | [M+H]^+^ |

## **Figure S1.8** HR-ESIMS spectrum of compound **1**

## **Figure S1.9** IR spectrum of compound **1**

## **Figure S1.10** DP4+ results of compound **1** (Isomer 1: 3*S**, 5*S**, 9*R**, 11*R**, 13*S**, 14*R**, 17*S**, 20*S**; Isomer 2: 3*S**, 5*S**, 9*R**, 11*R**, 13*S**, 14*R**, 17*S**, 20*R**).

| Nucleus | Parameter | Isomer 1 | Isomer 2 |
| --- | --- | --- | --- |
| ^13^C | R^2^ | 0.9978 | 0.9981 |
| ^13^C | MAE (ppm) | 1.821 | 1.765 |
| ^13^C | RMSD (ppm) | 2.620 | 2.500 |
| ^1^H | R^2^ | 0.8944 | 0.8768 |
| ^1^H | MAE (ppm) | 0.354 | 0.388 |
| ^1^H | RMSD (ppm) | 0.498 | 0.542 |

**Table S1.** Comparison of the NMR data around C-20 of compound **1** with those of **3** and **4**.

| **Compounds** | *δ*_H_, *δ*_C_ (ppm) | | | |
| --- | --- | --- | --- | --- |
|  | H-17, C-17 | H-21, C-21 | H-22, C-22 | H-23, C-23 |
| **1** | 1.92, 60.2 | **1.29**, 29.4 | 7.08, **157.6** | 6.33, 127.6 |
| **3** | 1.89, 60.7 | **1.28**, 29.4 | 7.05, **157.6** | 6.31, 127.3 |
| **4** | 1.87, 60.0 | **1.41**, 29.0 | 6.89, **152.6** | 6.28, 126.6 |

# 2. Spectra of compound 2

## **Figure S2.1** ^1^H NMR spectrum (600 MHz) of compound **2** in CD_3_OD

## **Figure S2.2** ^13^C NMR spectrum (150 MHz) of compound **2** in CD_3_OD

**Figure S2.3** DEPT spectrum (150 MHz) of compound **2** in CD_3_OD

## **Figure S2.4** HSQC spectrum (600 MHz) of compound **2** in CD_3_OD

## **Figure S2.5** HMBC spectrum (600 MHz) of compound **2** in CD_3_OD

## **Figure S2.6** ^1^H-^1^H COSY spectrum (600 MHz) of compound **2** in CD_3_OD

## **Figure S2.7** NOESY spectrum (600 MHz) of compound **2** in CD_3_OD

| Measured m/z | Cal m/z | Error(mmu) | Error(ppm) | Ion Formula | Ion |
| --- | --- | --- | --- | --- | --- |
| 431.2796 | 431.2791 | 0.5 | 1.0 | C26H39O5 | [M+H]^+^ |
|  |  |  |  |  |  |

## **Figure S2.8** HR-ESIMS spectrum of compound **2**

## **Figure S2.9** IR spectrum of compound **2**

## **Figure S2.10** DP4+ results of compound **2** (Isomer 1: 3*S**, 5*S**, 9*R**, 11*R**, 13*R**, 14*R**, 17*R**, 23*R**, 24*S**; Isomer 2: 3*S**, 5*S**, 9*R**, 11*R**, 13*R**, 14*R**, 17*R**, 23*S**, 24*R**; Isomer 3: 3*S**, 5*S**, 9*R**, 11*R**, 13*R**, 14*R**, 17*R**, 23*R**, 24*R**; Isomer 4: 3*S**, 5*S**, 9*R**, 11*R**, 13*R**, 14*R**, 17*R**, 23*S**, 24*S**)

# 3. Spectra of compound 3

## **Figure S3.1** ^1^H NMR spectrum (600 MHz) of compound **3** in CD_3_OD

**Figure S3.2** ^13^C NMR spectrum (150 MHz) of compound **3** in CD_3_OD

# 4. Spectra of compound 4

**Figure S4.1** ^1^H NMR spectrum (600 MHz) of compound **4** in CDCl3

**Figure S4.2** ^13^C NMR spectrum (150 MHz) of compound **4** in CDCl3

# 5. Spectra of compound 5

**Figure S5.1** ^1^H NMR spectrum (600 MHz) of compound **5** in CD_3_OD

**Figure S5.2** ^13^C NMR spectrum (150 MHz) of compound **5** in CD_3_OD

# 6. Spectra of compound 6

 **Figure S6.1** ^1^H NMR spectrum (600 MHz) of compound **6** in DMSO-*d*_6_

**Figure S6.2** ^13^C NMR spectrum (150 MHz) of compound **6** in DMSO-*d*_6_

# 7. Spectra of compound 7

 **Figure S7.1** ^1^H NMR spectrum (600 MHz) of compound **7** in CD_3_OD**Figure S7.2** ^13^C NMR spectrum (150 MHz) of compound **7** in CD_3_OD

# 8. Spectra of compound 8

**Figure S8.1** ^1^H NMR spectrum (600 MHz) of compound **8** in CD_3_OD

**Figure S8.2** ^13^C NMR spectrum (150 MHz) of compound **8** in CD_3_OD

# 9. Spectra of compound 9

**Figure S9.1** ^1^H NMR spectrum (600 MHz) of compound **9** in CD_3_OD

**Figure S9.2** ^13^C NMR spectrum (150 MHz) of compound **9** in CD_3_OD

# 10. Spectra of compound 10

**Figure S10.1** ^1^H NMR spectrum (600 MHz) of compound **10** in DMSO-*d*_6_

**Figure S10.2** ^13^C NMR spectrum (150 MHz) of compound **10** in DMSO-*d*_6_

# 11. Spectra of compound 11

**Figure S11.1** ^1^H NMR spectrum (600 MHz) of compound **11** in CD_3_OD

**Figure S11.2** ^13^C NMR spectrum (150 MHz) of compound **11** in CD_3_OD

# 12. ITS Sequence of *Lodderomyces* sp. Z-1

>Z-1_ITS

CCGCCTTATGATATGCTTAAGTTCAGCGGGTAATCCTACCTGATTTGAGGTCGAAGTTTGAAATATAGATTGGAGCTTTTATTAAGCACAATGGAGTGGTTAGACCTAATACATTGTTATAAATACTCCTCCTAACTTTCAAGCAAACCTGGCGTATTGCTCATCACCAAACCCGGGGGTTTGAGGGAGAAATGACGCTCAAACAGGCATGCCCTCCGGAATACCAGAGGGCGCAATGTGCGTTCAAAGATTCGATGATTCACGAATATCTGCAATTCATATTACTTATCGCATTTCGCTGCGTTCTTCATCGATGCGAGAACCAAGAGATCCGTTGTTGAAAGTTTTGACTATTAATAAATATAATCAGTTGACTAGTTTAAAATAAAAAATTTAGGTTGAGTTTATCCTCTGGCAGCAGAGCAATTAAGCAGCCACTGCCAAAGCAAGTTTTAAGAATAAAACAAAAACACGTGTGTAGATAAGAAAAGTGCAGTTAAGCACAATTCTCAAAATTCTGTAATGATCCTTCCGCAGGTTCACCCTAACCGGAAGTGT

# 13. NO Inhibition and Cell Viability

## **Table S2.** Inhibition of LPS-induced NO production and relative cell viability in RAW264.7 cells treated with compounds **1**–**11** at 50 μM

| Compounds | NO inhibition (%) | Relative cell viability (%) |
| --- | --- | --- |
| **1** | 13.69 ± 2.68 | 81.64 ± 3.80 |
| **2** | 25.61 ± 8.99 | 85.84 ± 1.45 |
| **3** | 8.83 ± 2.39 | 82.01 ± 0.83 |
| **4** | 13.91 ± 2.40 | 49.65 ± 2.10 |
| **5** | 9.93 ± 6.29 | 98.11 ± 0.65 |
| **6** | 7.28 ± 4.56 | 82.17 ± 2.57 |
| **7** | 10.15 ± 3.97 | 87.75 ± 0.38 |
| **8** | 26.27 ± 5.01 | 79.79 ± 1.46 |
| **9** | 12.80 ± 2.39 | 75.46 ± 2.21 |
| **10** | 61.81 ± 2.40 | 83.99 ± 2.42 |
| **11** | 7.76 ± 2.23 | 83.56 ± 1.55 |

*Note:* Compounds **1**–**11** were tested at 50 μM. Dexamethasone, used as the positive control, was tested at 20 μM and showed 65.56% ± 3.56% NO inhibition and 62.08% ± 2.36%relative cell viability. Cell viability was assessed using the CCK-8 assay under treatment conditions matching those of the NO-inhibition assay. Values represent the means ± SD of three technical replicate wells from a single experiment.
